# The novel developed microsatellite markers revealed potential hybridization among *Cymbidium* species and the interspecies sub-division of *C. goeringii* and *C. ensifolium*

**DOI:** 10.1186/s12870-023-04499-y

**Published:** 2023-10-13

**Authors:** Hui-Juan Ning, Fang-Fang Gui, En-Wei Tian, Li-Yuan Yang

**Affiliations:** 1 Zhejiang Provincial Key Laboratory of Germplasm Innovation and Utilization for Garden Plants, School of Landscape and Architecture, Zhejiang A&F University, Hangzhou, 311300 Zhejiang China; 2 School of Traditional Chinese Medicine, Southern Medical University, Guangzhou, 515005 China; 3 Guangdong Provincial Key Laboratory of Chinese Medicine Pharmaceutics, Guangzhou, 510515 China; 4 Key Laboratory of National Forestry and Grassland Administration On Germplasm Innovation and Utilization for Southern Garden Plants, Hangzhou, 311300 Zhejiang China

**Keywords:** SSR-Marker, RNA-Seq, *Cymbidium goeringii*, Inter-species sub-division, Hybridization

## Abstract

**Background:**

Orchids (*Cymbidium* spp.) exhibit significant variations in floral morphology, pollinator relations, and ecological habitats. Due to their exceptional economic and ornamental value, *Cymbidium* spp. have been commercially cultivated for centuries. SSR markers are extensively used genetic tools for biology identification and population genetics analysis.

**Result:**

In this study, nine polymorphic EST-SSR loci were isolated from *Cymbidium goeringii* using RNA-Seq technology. All nine SSR loci showed transferability in seven other congeneric species, including 51 cultivars. The novel SSR markers detected inter-species gene flow among the *Cymbidium* species and intra-species sub-division of *C. goeringii* and *C. ensifolium*, as revealed by neighborhood-joining and Structure clustering analyses.

**Conclusion:**

In this study, we developed nine microsatellites using RNA-Seq technology. These SSR markers aided in detecting potential gene flow among *Cymbidium* species and identified the intra-species sub-division of C*. goeringii* and *C. ensifolium*.

**Supplementary Information:**

The online version contains supplementary material available at 10.1186/s12870-023-04499-y.

## Background

Orchidaceae is one of the most abundant species angiosperm families, constitutes approximately 10% of flowering plant species, and displays unique flower morphologies [1–3]. Orchids account for a large share of the global floriculture trade both as cut flowers and as potted plants and were estimated to comprise around 10% of the international fresh-cut flower trade [4, 5]. Orchids are fast-growing potted flowering plants in many countries in terms of sales [6]. Hybridization between species happens in nature and during culturing [7, 8]. The genus *Cymbidium* comprises 44 species that are widely distributed in East Asia [9–11]. *Cymbidium* spp. (Orchidaceae) are popular potted flowers which were considered to have great value in ornamental and economic and have been cultivated for several centuries [12]. Despite the great value, the richness of orchid species decreased dramatically, and a lot of orchid species have become rare or endangered in the world [5, 13]. Because of a long history of cultivation and nature hybridization, the genetic variation of *Cymbidium* spp. is high diversity and complex [14]. Consequently, the taxonomic classification of *Cymbidium* becomes very difficult [15]. Although several approaches have been attempted to understand genetic diversity [16–19], the genetic resources for the characterization of *Cymbidium* are still insufficient. Some microsatellite markers that developed for the genus *Cymbidium* are not well-tested in cross-species [16, 17, 19]. Additionally, the genetic relationship among many of the major lineages of *Cymbidium* species remains unclear and the genetic relationship between species is not clear [9, 20]. It is necessary to develop reliable markers to evaluate the genetic diversity and phylogenetic relationship of *Cymbidium* for effective conservation and utilization.

Microsatellites or simple sequence repeats (SSRs) are a subcategory of tandem repeats consisting of 1–6 nucleotides in length (motifs) found in genomes of all prokaryotes and eukaryotes [21, 22]. Microsatellites have been utilized liberally over previous years since they are profoundly informative with a high mutation rate per generation per locus (10^−7^ to 10^−3^) [21] and relatively selective neutrality [23, 24] As high polymorphism, abundance, co-dominance, selective neutrality and transferability across species, microsatellite markers have been widely used in species and cultivars identification [25]. The availability of high-throughput sequencing technologies (RNA-Seq) has enabled researchers to identify a substantial number of microsatellites at less cost and effort compared to traditional SSR development processes [26].

In this study, nine novel microsatellite markers were developed and characterized based on RNA-Seq data. Combined with four SSR markers from published literature, thirteen SSR markers were used to figure out: (1) how prevalent these SSR markers are in cross-species amplification; (2) is there sub-division population structure intra-species; (3) is there genetic hybridization inter-species in the genus *Cymbidium.*

## Results

### Sequencing and de novo assembly of transcriptome

In total, 11.07 Gb of clean data was obtained using the Illumina NovaSeq platform. RNA-Seq yielded 22,739,372 clean paired-end reads at least 150 bp in length, and 72,556 Unigenes were gained from the clean reads performed by de novo assembly with Trinity. The average length of Unigenes is 835 bp. The N50 of the Unigenes was 1,483 bp.

### Unigenes annotation

**Fig. 1:**
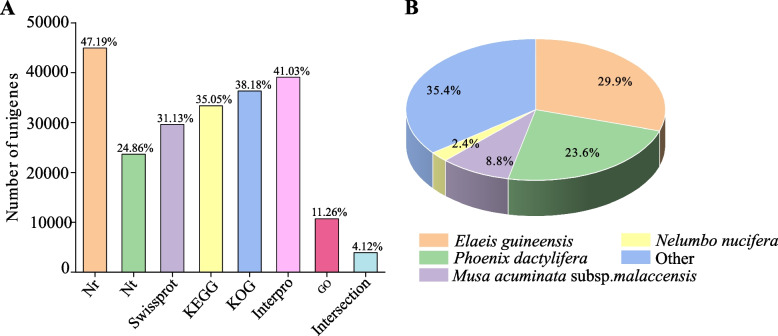
*Cymbidium goeringii* Unigenes annotation against eight databases (**A**) and against different species (**B**)

**Fig. 2:**
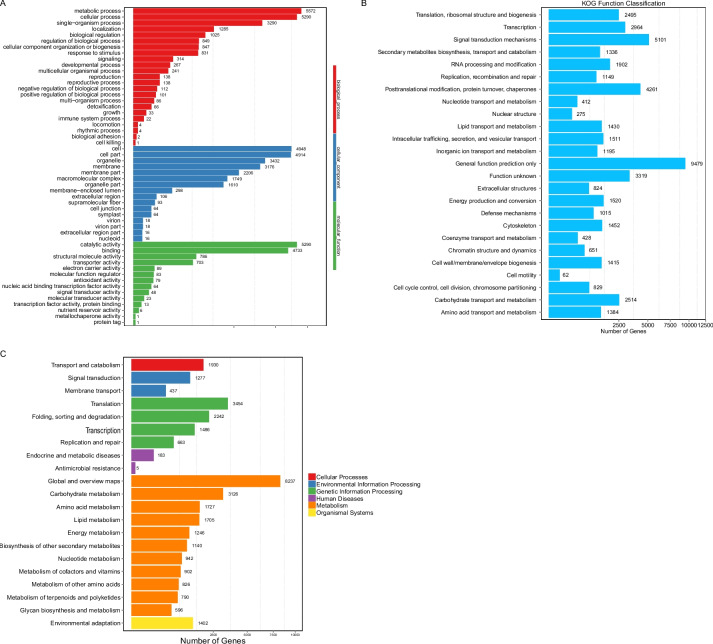
*Cymbidium goeringii* gene annotation based on GO, KOG, and KEGG databases. **A** Gene Ontology (GO) annotation graph of *C. goeringii*. **B** EuKaryotic Ortholog Groups (KOG) annotation graph of *C. goeringii*. **C** Kyoto Encyclopedia of Gene and Genomes (KEGG) annotation graph of *C. goeringii*

The assembled Unigenes of *C. goeringii* were annotated against eight public databases (Table 1, Fig. 1A). A total of 49,636 Unigenes (42.13%) were successfully annotated against at least one database. In summary, 29.86% of Unigenes from *C. goeringii* were matched with *Elaeis guineensis*, and 23.56% matched *Phoenix dactylifera* (Fig. 1B). In total, 10,724 Unigenes (11.26%) were annotated and clustered into three main GO categories and 50 sub-categories (Fig. 2A). Based on the KOG database, 36,364 Unigenes were annotated and 19.37% of Unigenes were annotated into the ‘General function’ cluster (Fig. 2B). Based on the KEGG database, a total of 33,377 Unigenes were annotated (Fig. 2C).

**Table 1:** Unigenes annotation of *C. goeringii* against eight databases

Annotation database	Number of Unigenes	Percentage (%)
Annotated in NR	44,934	47.19%
Annotated in NT	23,672	24.86%
Annotated in KEGG	33,377	35.05%
Annotated in SwissProt	29,641	31.13%
Annotated in Interpro	39,072	41.03%
Annotated in GO	10,724	11.26%
Annotated in KOG	36,354	38.18%
Annotated in Intersection	39,072	41.03%
Annotated in at least one database	49,636	52.13%
Total Unigenes	95,224	100%

### Frequency and distribution of SSRs in the transcriptome

**Fig. 3:**
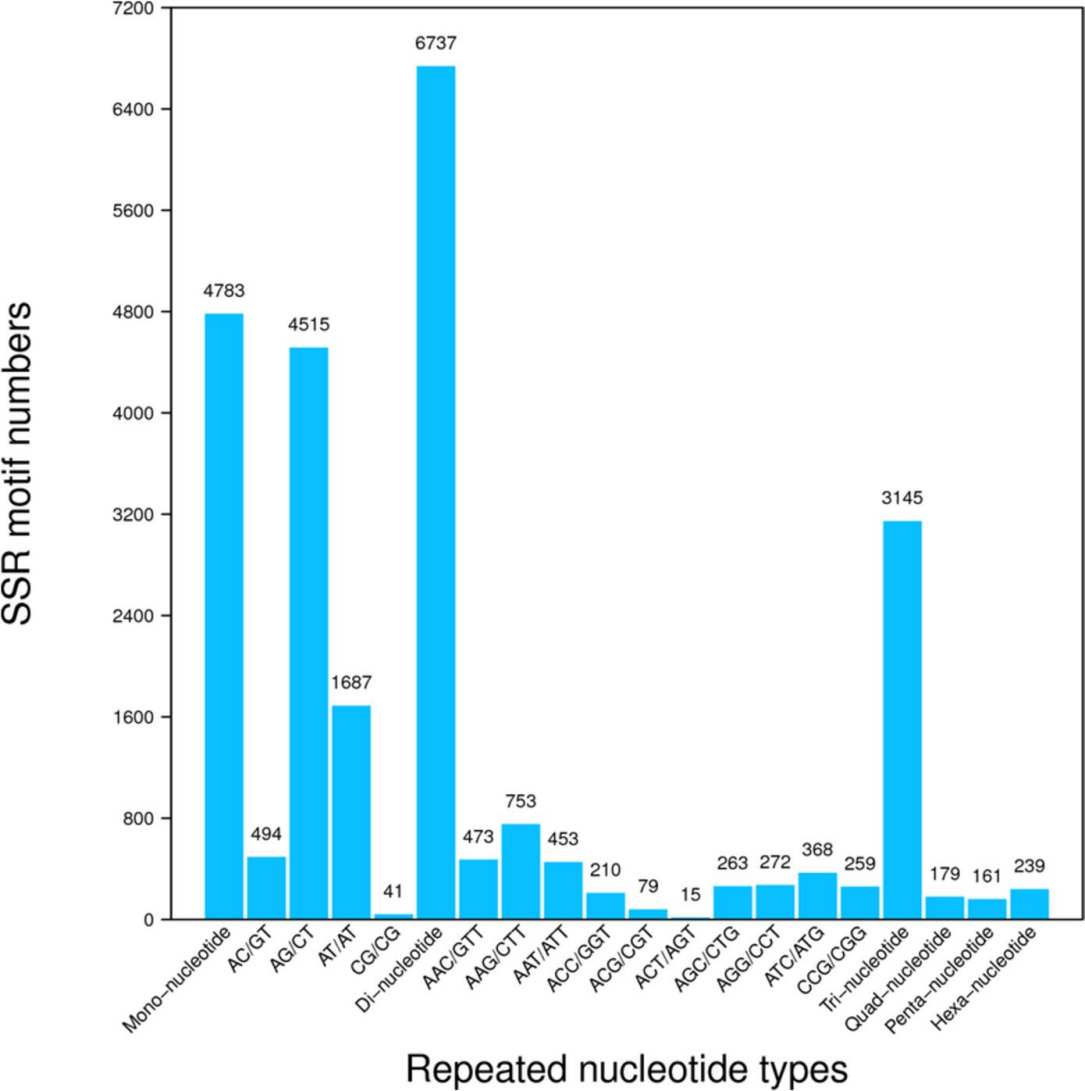
Microsatellite loci distribution in the transcriptome data of *C. goeringii*

Using the MISA software, a total of 95,224 Unigenes were scanned and 15,244 SSR loci were detected (Table 2). The SSR locus discovered from transcriptome data includes six types: mono-, di-, tri-, tetra-, penta-, and hexanucleotide repeat motifs. The content among different types varies greatly. The Di-nucleotide repeat motif ranked the most abundant type (accounting for 44.19%) and the penta-nucleotides was the least abundant type (accounting for 1.11%) (Fig. 3). The counts of four types of Di-nucleotide and ten types of Tri-nucleotide were presented at Fig. 3.

**Table 2:** Prediction of SSRs out of the transcript datasets of *C. goeringii*

Item	Number
Total number of sequences examined	95224
The total size of examined sequences (bp)	79602818
Total number of identified SSRs	15244
Number of SSR-containing sequences	12745
Number of sequences containing more than one SSR	2051
Number of SSRs present in compound formation	962
Mono nucleotide	4783
Di nucleotide	6737
Tri nucleotide	3145
Tetra nucleotide	179
Penta nucleotide	161
Hexa nucleotide	239

### Genetic polymorphisms of 13 SSR loci

In total, nine loci were selected from the transcriptome data of *C. goeringii*. The sequences of the nine loci were submitted to NCBI (https://www.ncbi.nlm.nih.gov/nuccore/OP480183-OP480192) (Table 3). Combined with the four loci from published literature [17, 27], 167 alleles were detected from *C. goeringii* population (Table 3). The observed heterozygosities varied dramatically cross 13 loci, ranged from 0.09 to 1. The expected heterozygosities of most loci were lager than 0.75, only locus H93 present the observed heterozygosities of 0.54. Null allele frequency ranged from 0 to 0.8 across 13 SSR markers (Table 4). Linkage disequilibrium was not detected between any pair of loci.

**Table 3:** Characteristics of 9 microsatellite loci isolated from *Cymbidium goeringii*

**Locus**	**Motif**	**Primer sequence (5’- 3’)** **Forward/ reverse**	Ta(℃)	Product Size range (bp)	GenBank accession no
LH3	(TTA)_6_	TTTCTTGCTGAGCCTTTTATGTC/CCACTCCTTTTCTTCATCATTTG	54	118–151	OP480182
LH4	(AG)_7_	AAAAATAGGCATAGTCGTCCGTC/TCTTATTTTATCCGGGGAGAGC	54	132–168	OP480183
LH6	(CT)_26_	ACTCGACTCGACTCACTTCAAAA/AAGTTAAATAACCCCACCAGCAC	54	121–149	OP480184
LH14	(AG)_6_	AGCTTGATGAAATTGCTGAAAAG/GGCAAAGGATCTGTATTCTTCCT	54	107–109	OP480186
LH33	(CT)_6_	CTATCTGCAGTGTTTCTCAAGCA/CGCAATACCTCGATACCAAATAC	54	159–189	OP480188
LH43	(CT)_15_	TTAAATTCAAAGTTTCACTCGCC/AACTCCCCAGTAGCTTTCAGTTT	54	129–167	OP480189
LH45	(GCA)_5_	CTTTCTTCGCGCATATGTACTTT/ATCAAAGCTCACCATTTGTTCAT	54	123–162	OP480190
LH88	(TA)_8_	AACTACAGCTTCATATTTGGCGA/GCTATGATTCCCCTTTTTCAATC	54	112–142	OP480191
LH93	(TCC)_5_	TTCCACAATAGTTCCCCTGTCTA/AGGAAACAGAGGAAGAGGAAGAA	54	84–96	OP480192

**Table 4:** Genetic diversity 13 SSR markers based on *C. goeringii*

Locus	Product size(bp)	*Na*	*Ne*	*f*_*NA*_	*I*	*PIC*	*Ho*	*He*	*F*
L2	178–298	13	8.4	0.32	2.30	0.70	0.38	0.88	0.57
L3	336–392	16	9.3	0.38	2.48	0.93	0.22	0.89	0.76
X1	177–241	11	5.1	0.09	2.04	0.84	0.67	0.80	0.17
X2	130–190	6	4.2	0.39	1.56	0.91	0.09	0.76	0.88
LH3	118–160	8	4.8	0.15	1.79	0.86	0.50	0.79	0.37
LH4	107–207	22	14.6	0.06	2.88	0.92	0.83	0.93	0.11
LH6	110–160	21	15.8	0.00	2.89	0.94	1.00	0.94	-0.07
LH14	106–122	5	2.6	0.16	1.17	0.82	0.54	0.62	0.13
LH33	158–212	15	9.4	0.22	2.44	0.86	0.57	0.89	0.37
LH43	124–192	22	16.3	0.02	2.94	0.94	0.86	0.94	0.09
LH45	123–163	10	5.4	0.19	1.91	0.91	0.54	0.81	0.33
LH88	101–163	13	6.5	0.30	2.14	0.90	0.29	0.85	0.66
LH93	63–93	5	2.2	0.80	1.08	0.85	0.04	0.54	0.92

*Na* number of alleles, *Ne* Effective number of alleles, *f*_*NA*_ null-allele frequency, *I* Shannon's information index, *PIC* Polymorphism information content, *F* Fixed index, *H*_O_ observed heterozygosity, *H*_E_ expected heterozygosity

### Cross-species analysis

**Fig. 4:**
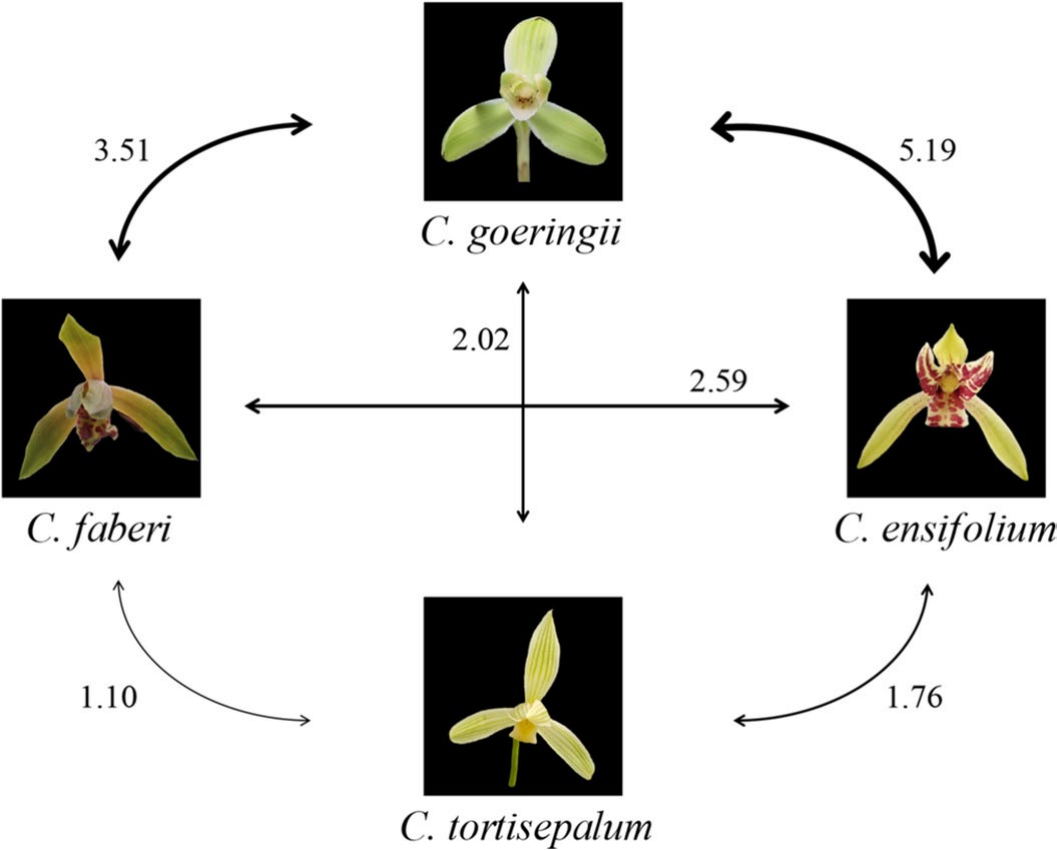
Gene flow among *C. faberi*, *C. ensifolium*, *C. tortisepalum*, and *C. goeringii*. The number noted on the line between species indicated the number of migrants (Nm) between species

The 9 polymorphic SSR loci isolated from *C. goeringii* (Table 3) and 4 loci from published literature (Table S1) were tested for cross-species amplification with 72 individuals from eight *Cymbidium* species. all these loci could be successfully amplified across eight *Cymbidium* species (Table 5). The genetic diversity for the eight *Cymbidium* species were listed in Table 5. The gene flow between species were presented in Fig. 4. Strong gene flow was detected between *C. goeringii* and *C. ensifolium* (Nm = 5.19). *C. goeringii* and *C. faberi* also present strong inter-species gene flow (Nm = 3.51). *C. tortisepalum* presents weak gene flow with other species (Fig. 4).

**Table 5:** Genetic diversity of eight *Cymbidium* species

Species	Sample size	*Nps*	*Na*	*Ne*	*I*	*Ho*	*He*
*C. longibracteatum*	2	2.38	2.25	0.75	0.54	0.45	-0.19
*C. goeringii*	21	11.77	7.47	2.04	0.52	0.81	0.38
C. *serratum*	2	2.23	2.18	0.69	0.23	0.44	0.57
*C. kanran*	3	3.92	3.42	1.22	0.58	0.65	0.18
*C. faberi*	16	8.00	5.25	1.57	0.35	0.69	0.55
*C. ensifolium*	17	10.08	6.11	1.90	0.44	0.78	0.46
*C. tortisepalum*	10	7.23	5.16	1.59	0.45	0.68	0.31
*C. sinense*	1	1.54	1.54	0.37	0.54	0.27	-1.00

*Nps* Number of provenance samples, *Na* number of alleles, *Ne* Effective number of alleles, *I* Shannon's information index, *H*_O:_ observed heterozygosity, *H*_E_ expected heterozygosity

### Principal coordinate (PCoA) analysis of four *Cymbidium* species

**Fig. 5:**
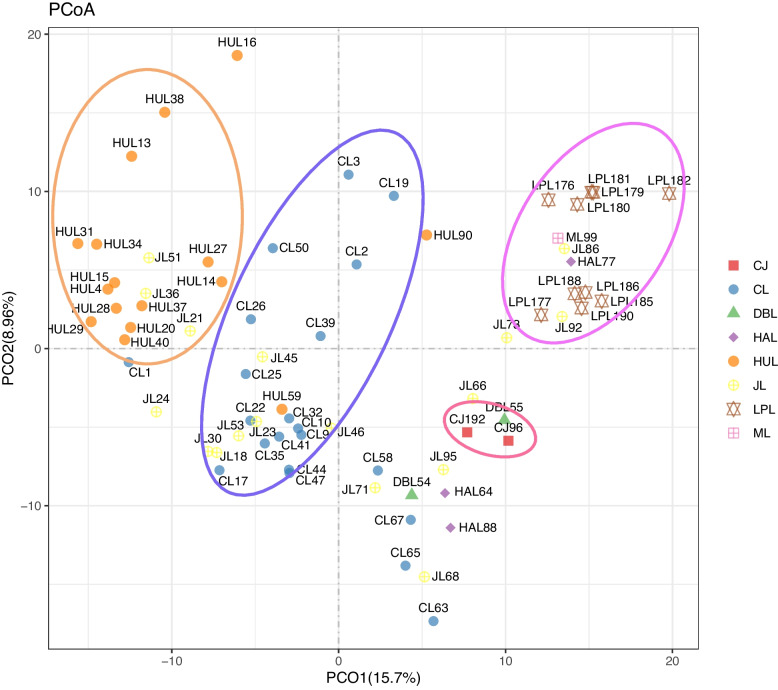
PCoA analysis of the 72 individuals from 8 species of *Cymbidium*. HUI: *C. faberi*; JL:*C. ensifolium*; ML:* C. sinense*; HAL:* C. kanran*; CJ:* C. longibracteatum*; LPL:* C. tortisepalum*; DBL: *C. serratum*; CL: *C. goeringii*

In Principal coordinate (PCoA) analysis, the first two principal component accounts for 24.66% (Fig. 5). Most *C. tortisepalum* (LPL) individuals clustered together and most *C. faberi* (HUL) individuals clustered together. Two *C. longibracteatum* (CJ) individuals clustered together. But *C. goeringii* (CL) and *C. ensifolium* (JL) individuals are scattered (Fig. 5).

### The Neighbor-Joining phylogenetic analysis

**Fig. 6:**
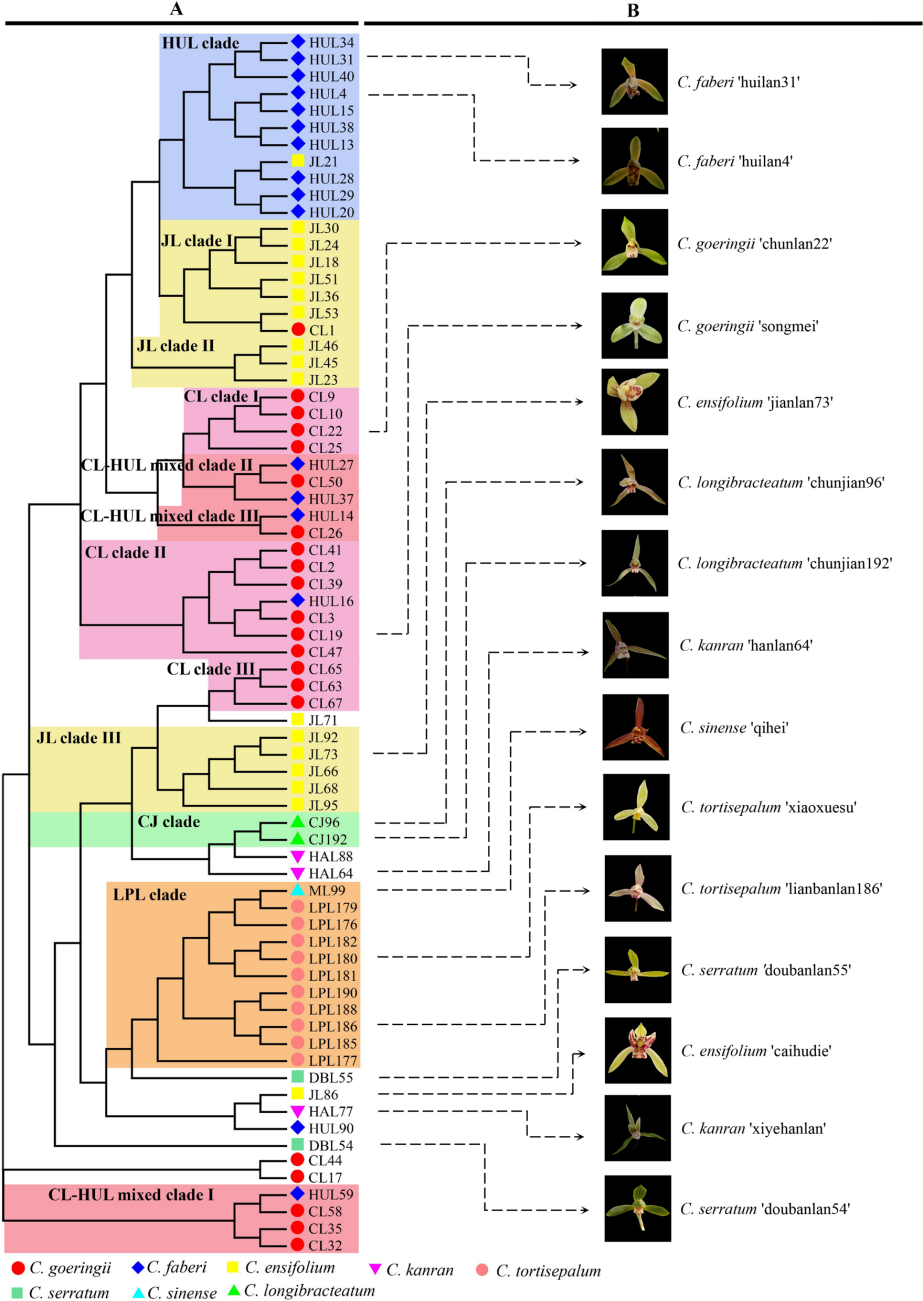
Neighbor-joining phylogenetic tree of 8 *Cymbidium* species. 72 individuals were included in the phylogenetic analysis. **A** phylogenetic tree. **B** partial pictures of the cultivars

Based on the distance calculation method of Shared Allele, the Neighbor-Joining phylogenetic analysis presented the phylogenetic relationship of the 72 *Cymbidium* individuals (Fig. 6). Most of the individuals belong to the same species clustered together in the Neighbor-Joining tree. Such as all the *C. tortisepalum* individuals cluster into the LPL clade; ten *C. faberi* individuals clustered in the HUL clade and most *C. goeringii* and *C. ensifolium* individuals clustered in CL clades and JL clades separately (Fig. 6).

But intra-species sub-division existed in both *C. ensifolium* (JL) and *C. goeringii* (CL), and each species contains three sub-clades (Fig. 6). The Neighbor-Joining tree also revealed some gene flow between *Cymbidium* species. For example, one *C. goeringii* (CL1) individual was mixed into *C. ensifolium* sub-clade (JL clade I); one *C. sinense* (ML99) individual was mixed into the *C. tortisepalum* (LPL) clade, and one *C. ensifolium* individual (JL 21) was mixed into *C. ensifolium* clade (HUL clade) Multiple mismatches exist between *C. ensifolium* and *C. goeringii*. One *C. ensifolium* individual (HUL 16) was mixed into *C. goeringii* sub-clade (CL clade II). There were multiple *C. ensifolium* and *C. goeringii* individuals clustered together and constituted mixed clades, such as CL-HUL mixed clade in Fig. 6.

### The population structure analysis

**Fig. 7:**
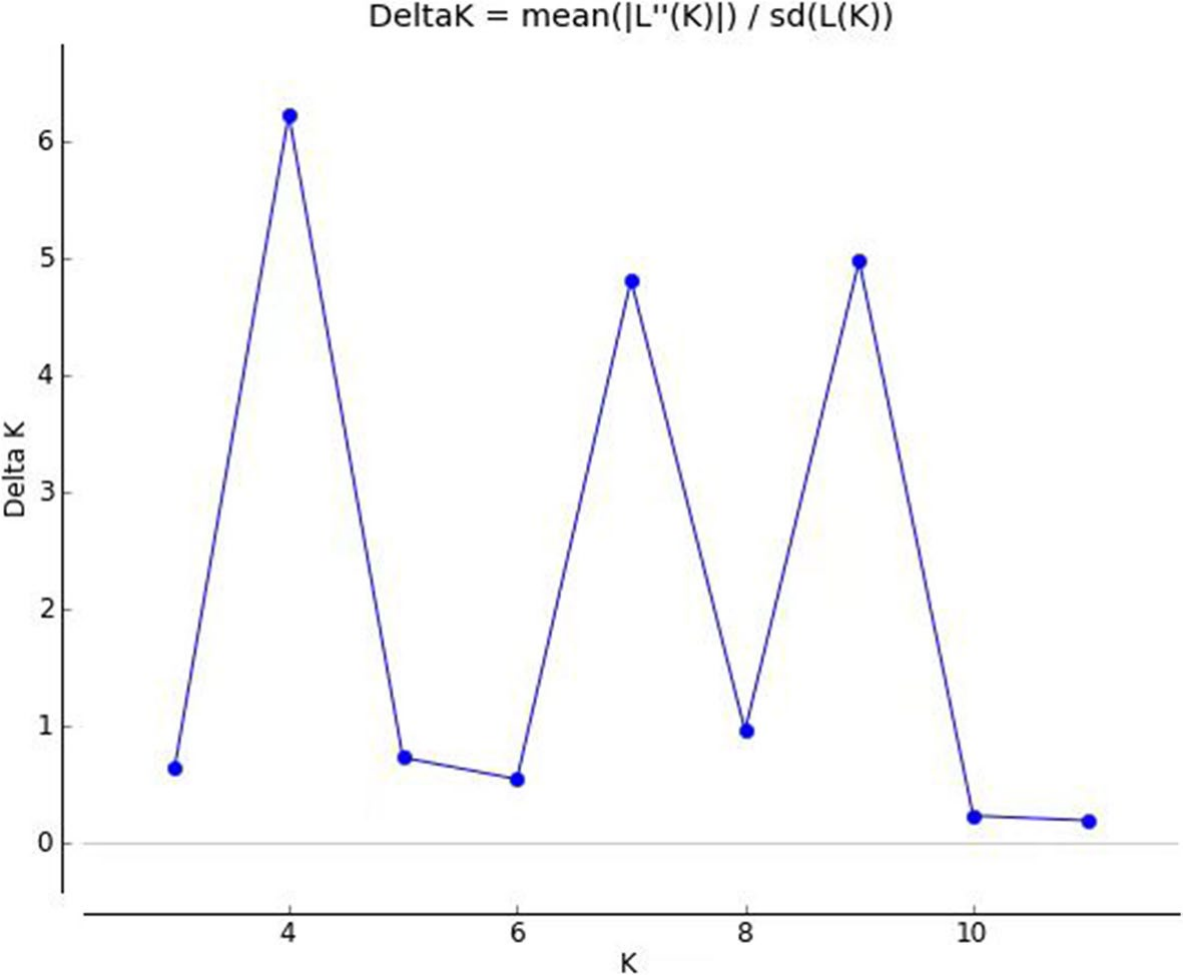
the magnitude of DeltaK (B) as a function of K suggested the existence of 4 clusters for *Cymbidium.* Results are from 10 replicates for each of 1 ≤ K ≤ 9

**Fig. 8:**
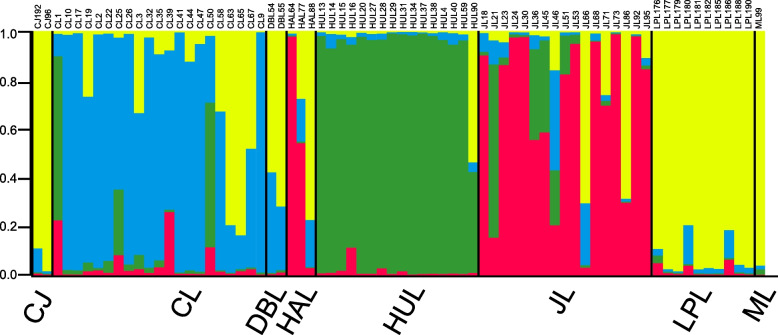
STRUCTURE genetic clusters of 72 individuals (up) and 8 species (down) of genus *Cymbidium*. Green, yellow, blue, and red represent the assignment probability for the four major clades

In the population structure analysis, the magnitude of Delta K as a function of K suggested the existence of 4 clusters for *Cymbidium.* when K = 4, the value Delta K was the largest (Fig. 7). We present the structure result when K = 4 (Fig. 8). The 4 clusters were presented using 4 colors: yellow, green, red, and blue, and the percentage of each color presented the proportion of each cluster individually. The yellow cluster takes more than 90% of most *C. tortisepalum* (LPL) individuals. *C. tortisepalum* was mainly constituted by a yellow cluster. Only one *C. sinense* individual (ML 99) was included in this work, and the was constituted mainly by yellow which is very similar to the constitution of *C. tortisepalum*. *C. faberi* was mainly constituted by green clusters. The green cluster was also contained in *C. goeringii* and *C. ensifolium.* The color constitution of *C. goeringii* and *C. ensifolium* were very complex. Both *C. goeringii* and *C. ensifolium* contain four clusters in Structure analysis (Fig. 8).

## Discussion

### The novel developed microsatellite markers by transcriptome sequencing for *Cymbidium*

In this study, nine *Cymbidium* SSR markers were developed using the RNA-seq technique. The availability of high-throughput sequencing technologies has recently assisted researchers, providing excellent opportunities for life sciences [28]. Generating transcriptome data through RNA sequencing has been successfully reported for SSR marker development in non-model plants with no reference genome as de novo sequencing [29–32]. Compared with the SSRs developed from genomic sequences, the SSR markers isolated from transcripts (ETS-SSR) displayed high transferability among related species and high genetic differentiation, low error rates, and low null allele frequencies but relatively low polymorphisms [33]. In this study, the transcriptome data provide abundant resources of the SSR sites, which would be useful in studies on the genetic diversity, and population genetics of *C. goeringii* and congeneric other species.

In this study, the newly developed microsatellite markers are highly transferable in the genus *Cymbidium*. The nine SSR loci could be successfully amplified across eight *Cymbidium* species (Table 5). Microsatellite was one of the most widely used neutral molecular markers [21–24]. Because of the high level of polymorphism, high abundance, co-dominance, selective, neutrality, and transferability across species, microsatellite markers have been utilized for a variety of applications in plant studies, including species/cultivars identification, paternity testing, genes mapping, construction of linkage maps, markers assisted selections and back-crosses, population genetics, gene flow, phylogenetics, and conservation genetics [25, 34, 35]. The nine microsatellite markers could be successfully amplified in eight *Cymbidium* species, and proforma highly polymorphic. Up to 22 alleles were detected in two loci (Table 4). As the urgent need for an identification method in orchid business marketing, our newly developed microsatellite markers will be useful in *Cymbidium* species and cultivars discrimination and identification both in orchid business and research.

### The intra-species sub-division

Phylogenetic analysis was frequently used in resolving the genetic variation and structure of Orchidaceae species [36–40]. Using the novel developed SSR markers, the population genetic analysis in the genus *Cymbidium* revealed intra-species divergence and inter-species hybridization. The phylogenetic analysis presented the intra-species divergence in both *C. ensifolium* (JL clade I, JL clade II and JL clade III) and *C. goeringii* (CL clade I, CL clade II and CL clade III) species (Fig. 6). In the PCoA analysis, unlike *C. tortisepalum* and *C. faberi*, in which individuals clustered together, the *C. goeringii* and *C. ensifolium* individuals are scattered (Fig. 5). The intra-species divergence was also presented in the STRUCTURE analysis (Fig. 8). The cultivators (individuals) of *C. goeringii* and *C. ensifolium* presented complex constitutions. In natural populations, *C. ensifolium* and *C. goeringii* present low-level genetic diversity between populations [41, 42]. In this work, the genetic structure is more significant than the natural population. That may be the consequence of artificial breeding accelerated genetic divergence. In this work, all the *C. ensifolium* and *C. goeringii* individuals are cultivators. Genetic diversity analysis discovered more genetic divergence in cultivators. Using RAPD markers, two distinct groups were revealed among cultivators of *C. goeringii* [20]. Based on 38 *C. ensifolium* cultivars, high genetic diversity was discovered using RAPD analysis [43]. This indicated that higher genetic diversity exists in the cultivator than in the natural population of *C. ensifolium* and *C. goeringii.*

### Inter-species gene flow among *Cymbidium* species

Neutral molecular markers were frequently used in detecting inter-species hybridization and gene flow [24, 34, 44]. In this work, based on SSR markers, using PCoA and phylogenetic analysis high-frequency gene flow was detected among *C. goeringii*, *C. ensifolium*, and *C. faberi*. High number of migrants (Nm) was detected among *C. goeringii*, *C. ensifolium*, and *C. faberi* (Fig. 4). In the PCoA analysis, two individuals were clustered into *C. goeringii* clade and one *C. goeringii* individual was clustered into *C. faberi* clade in (Fig. 5). Multiple *C. ensifolium* individuals were clustered into *C. goeringii* clade or *C. goeringii* clade in PCoA analysis (Fig. 5). In phylogenetic analysis, multiple *C. goeringii* and *C. faberi* individuals clustered together and formed mixed clades in NJ analysis (Fig. 6). One *C. goeringii* (CL 1) individual was clustered into *C. ensifolium* (JL clade I), one *C. ensifolium* (JL 21) individual was clustered into *C. faberi* (HUL clade) (Fig. 6). All these evidences indicated gene flow between *C. goeringii* and *C. faberi*.

Gene flow between *Cymbidium* species was not discovered for the first time. In one molecular genetic analysis work on the genus *Cymbidium*, one *C. faberi* cultivator ‘Ruyisu’ was clustered into *C. goeringii* group in STRUCTURE analysis based on SSR markers [17]. Sympatric distribution may cause inters-species hybridization in Orchid. The natural distribution of *C. goeringii* and *C. faberi* overlaps frequently, and both distributes in southwest and southeast China [45]. Sympatric distributed interspecific hybridization was discovered in another genus of Orchid. Natural hybridization were detected and proved between sympatric distributed *Geodorum eulophioides* and *G. densiflorum* [46].

Artificial cross-breeding may be another reason for the inter-species gene flow in *Cymbidium.* Orchids have been cultivated for centuries, artificial cross-breeding in *Cymbidium* is quite frequency [47], and hybridization between species happens multiple times during culturing [7, 8, 12]. In this work, three cultivators of *C. goeringii* and *C. faberi* were clustered into CL-HUL mixed clade (Fig. 6), and indicated complex genetic background of these three cultivators (Fig. 6).

## Conclusions

The newly developed microsatellite markers of *Cymbidium goeringii* with RNA-seq data were highly polymorphic, and successfully amplified across 8 *Cymbidium* species. Based on the SSR markers, intra-species sub-division was detected in *C. goeringii* and *C. ensifolium*; inter-species gene flow was detected among *C. goeringii*, *C. ensifolium*, and *C. faberi.* These SSR makers will be useful in the genus *Cymbidium*'s cultivar and species identification and population genetic cultivar*.*

## Methods

### Materials

Fresh leave of *Cymbidium goeringii* ‘da fu gui’ was collected for RNA extraction and transcriptome sequencing. *C. goeringii* ‘da fu gui’ was a popular orchid cultivator and classic representative of spring orchids with lotus petal flowers. *C. goeringii* ‘da fu gui’ was collected from natural forests in 1909. The transcriptome sample used in this experiment was initially brought from the seedling and plant company at Shaoxing, Zhejiang Province, China, and then cultured at the Orchid greenhouse of Zhejiang A&F University by Dr. Hui-Juan Ning.

In total, 72 individuals from 8 *Cymbidium* species were collected for the SSR marker screening experiment. Including 21 *C. goeringii* individuals,16 *C. faberi* individuals, 17 *C. ensifolium* individuals, 10 *C. tortisepalum* individuals, 2 *C. longibracteatum* individuals, 2 *C. serratum* individuals, 3 *C. kanran* individuals, and 1 *C. sinense* individual (Table 5). All of these *Cymbidium* specimens were collected from southeast and southwest China (Table S2) and identified by Dr. Hui-Juan Ning (the author of this work) and preserved at the Orchid greenhouse of Zhejiang A&F University. The detail of the collection location, the cultivars’ name, and the morphology of all the samples were listed in supplementary table 1. The specimens used in this work were purchased from plant companies and these 8 species have not been listed in national key protected plants. We collected the samples without any required permissions. Our sample collection work and experimental research complied with local legislation and national and international guidelines. All the plant materials were cultured at the Orchid greenhouse of Zhejiang A&F University (ZAFU) or persevered deposited at the herbarium of ZAFU. The voucher no. of each specimen was listed in Table S2.

### DNA extraction, RNA extraction, cDNA library construction and sequencing

The total RNA of one *C. goeringii* individual was extracted using a modified CTAB RNA extraction method for further transcriptome sequencing [48]. The genomic DNA of all the specimens was extracted using a modified DNA extraction method to detect polymorphisms of isolated microsatellite loci [49]. The quality and quantity of the exacted DNA and RNA was assessed using 1.5% agarose gel electrophoresis and NanoDrop 2000 spectrophotometer (Thermo Scientific, Wilmington, DE, USA). RNA-Seq library was constructed using Illumina TruSeq RNA Sample Preparation Kit (Illumina, San Diego, California, USA). The *C. goeringii* RNA was sequenced with RNA-Seq on the Illumina NovaSeq platform at BGI Tech (Shenzhen, China) generating 6.8 Gb reads.

### Transcriptome assembly and Unigenes annotation

The raw data yielded from RNA-Seq was conducted through a quality assessment and credibility analysis using Trimmomatic [50]. Low-quality sequences were removed in the sequencing process. Trinity was used for conducting the de novo assembly [51, 52]. The transcripts were assembled and the main transcript was selected from the local area as Unigenes [53].

Unigenes sequences were compared against NCBI nr (National Center for Biotechnology Information non-redundant protein sequences), NT (Nucleotide Sequence Database) KOG (EuKaryotic Orthologous Groups of proteins), SwissProt (Swiss-Prot Sequence Database), KEGG (the Kyoto Encyclopedia of Genes and Genomes), Intersection, and Interpro databases to associate Unigenes with annotated proteins and functional information [54–56]. Gene ontology analyses were conducted using Blast2GO [57]. WEGO [58] was used to characterize GO annotations and statistics, and to describe the molecular functions of genes, cell components, and biological processes involved.

### Microsatellites identification based on transcriptome data

The microsatellite tool (MISA-web) [59] was conducted to detect microsatellite loci with the following criteria [29]: mono-nucleotide repeat motifs with at least 12 repeats, di-nucleotide repeat motifs with at least six repeats and repeats of all other motif lengths extend at least five repeats.

Based on the Unigenes, SSR primers were designed using Primer Premier v5.0 software [60]. After primer designing, 120 pairs of primers were randomly selected with the condition of having targeted product sizes between 100 and 300 bp [29, 61]. Di-, tri-, tetra-, penta-, and hexanucleotide repeat loci have at least 9, 6, 5, 4, and 3 repeats, respectively.

### PCR amplification and genotyping

Twenty-one *C. goeringii* individuals were amplified to survey the polymorphism of the SSR loci. PCR amplification was performed under an appropriate annealing temperature (Table 2).The primers were attached FAM or HEX fluorescent (Applied Biosystems, New York, USA). Fragment sizes were determined on an ABI 3100 Genetic Analyzer (Applied Biosystems). ROX 500 (Applied Biosystems) was used as the internal lane size standard.

### SSR markers data analysis and cross-species analysis

GenALEX [62] was used to calculate the number of alleles (*Na*), the effective number of alleles (*Ne*), Shannon's information index (*I*), PIC Polymorphism information content (*PIC*), and the Fixed index (*F*) of each locus based on the data of *C. goeringii*. The likelihood ratio test was employed to estimate linkage disequilibrium using Genepop [63] and *P*-values were adjusted using the Bonferroni correction. The null-allele frequency was analyzed using Genepop [63].

To validate the transferability of the polymorphic loci isolated from *C. goeringii*, cross-species amplifications were tested for the 72 individuals from eight *Cymbidium* species using the same procedures as above, except that the annealing temperature was re-optimized for each locus. The number of provenance samples (*Nps*), number of alleles (*Na*), effective number of alleles (*Ne*), Shannon's information index (*I*), observed heterozygosity (*Ho*), and expected heterozygosity (*He*) was calculated for each species using GenALEX [62]. The pairwise species estimates of the number of migrants (Nm) were calculated among *C. goeringii*, *C. ensifolium*, *C. tortisepalum*, and *C. faberi* using GenALEX [62].

### Cluster analysis of eight *Cymbidium* species

GenALEX [62] was used to calculate the Pairwise Population Matrix of Nei Genetic Identity between populations, followed by PcoA analysis using the Omic share website tool (https://www.omicshare.com/tools/).

Powermarker software [64] was used to calculate the genetic distance based on the Shared Allele algorithm, and then a phylogenetic tree was constructed based on the Neighbor-Joining method, and the final results were visualized with MEGA version X [65].

The population structure analysis was performed using Structure v2.3.4 [66], the parameters length of the burn-in period was set to 100,000 and the number of MCMC Reps after burn-in was set to 500,000, the optimal K value was calculated using the harvest online website (https://taylor0.biology.ucla.edu/struct_harvest/), then repeated sampling analysis was performed with CLUMPP [67], visualization was performed with distruct software [66].

### Supplementary Information

**Additional file 1: Supplementary Table S1.** Characteristics of 4 microsatellite loci isolated from published literature. **Supplementary Table S2.** Sample collection of 72 individuals from 8 *Cymbidium* species.

## Data Availability

The datasets generated and/or analyzed during the current study are available in the NCBI (https://www.ncbi.nlm.nih.gov/nuccore/OP480183-OP480192).
